# Continent‐wide evidence that landscape context can mediate the effects of local habitats on in‐field abundance of pests and natural enemies

**DOI:** 10.1002/ece3.9737

**Published:** 2023-01-11

**Authors:** Salma Akter, Syed Z. M. Rizvi, Ahsanul Haque, Olivia L. Reynolds, Michael J. Furlong, Maria C. Melo, Terry Osborne, Jianhua Mo, Simon McDonald, Anne C. Johnson, Geoff M. Gurr

**Affiliations:** ^1^ School of Agricultural, Environmental and Veterinary Sciences Charles Sturt University Orange New South Wales Australia; ^2^ Gulbali Institute Charles Sturt University Orange New South Wales Australia; ^3^ Department of Entomology Sher‐e‐Bangla Agricultural University Dhaka Bangladesh; ^4^ Applied BioSciences Macquarie University Sydney New South Wales Australia; ^5^ Bio‐production Loam Bio Orange New South Wales Australia; ^6^ Susentom Victoria Heidelberg Australia; ^7^ Institute of Applied Ecology and Research Centre for Biodiversity and Eco‐Safety Fujian Agriculture and Forestry University Fuzhou China; ^8^ School of Biological Sciences, The University of Queensland Brisbane Queensland Australia; ^9^ New South Wales Department of Primary Industries Yanco Agricultural Institute, Private Mail Bag Yanco New South Wales Australia; ^10^ The Spatial Data Analysis Network Albury New South Wales Australia

**Keywords:** adjacent habitats, arthropods, brassica vegetables, in‐field positions, landscape structure

## Abstract

1. Landscape‐scale factors known to influence in‐field abundance of pest herbivores and their natural enemies, but little is known about effects that operate through the shorter‐range influences exerted by habitats immediately adjacent to crop fields.
2. This study first compared the abundance of brassica insect pests and their natural enemy arthropods in 24 spatially independent brassica vegetable fields across southern Australia. An ‘edge effect’ index was used to compare the abundance of each taxon in the field center with abundance in areas of the crop adjacent to differing habitats. Then, three landscape properties: landscape composition, edge density, and connectivity of diverse crop and non‐crop habitats were analyzed at five scales up to 5 km from these focal field centers to assess longer‐range influences on arthropod abundances in field centers and on the edge effects.
3. Edge effect of adjacent woody vegetation promoted ladybirds and reduced diamondback moth and whiteflies. Conversely, the presence of crops and pastures immediately adjacent to focal crop fields reduced whiteflies and aphids but with no effect on natural enemies.
4. Effect of landscape composition and connectivity on arthropod abundance at field center found promotion of aphids (cabbage aphid and green peach aphid) by woodland in the landscape.
5. Effect of landscape properties on the edge effects of adjacent habitats was contrasting; strengthened (landscape composition and edge density on edge effect of crops, pasture and woody vegetation in reducing diamondback moth and whiteflies) as well as weakened (edge density and landscape connectivity on edge effect of crops, pasture and woody vegetation in reducing diamondback moth on diamondback moth, whiteflies and aphids, and promoting ladybirds).
6. *Synthesis and applications*: Findings of this geographically extensive study help define the level of pest risk associated with sites as well as suggest potential interventions such as establishment or restorations of woody vegetation adjacent to crop fields that could reduce risk.

## Graphical abstract

This article attempted to investigate the longer and shorter range effects of landscapes on the in‐field abundance of pests and natural enemies in brassica vegetable fields. Although woody vegetation can enhance natural enemy abundance in the fields, the risk of these habitats in enhancing pest insects needs to be considered in the future pest management plan.

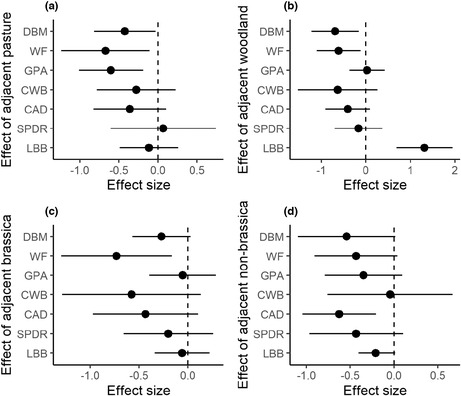

## 1 INTRODUCTION

Agricultural intensification reduces habitat diversity in farming landscapes (Tscharntke et al., 2005). Protecting or restoring landscape diversity can enhance the abundance of natural enemies and biological pest suppression in crop fields (Tscharntke et al., 2021). Importantly, however, while non‐crop habitats can benefit natural enemies (Blitzer et al., 2012; Perović et al., 2010), this is not always the case (Karp et al., 2018). Indeed, enhanced pest abundance can result even with a high proportion of non‐crop habitats in the landscape (Plećaš et al., 2014). Karp et al. (2018) proposed that disregarding the influence of local scale properties may be one of several possible reasons for inconsistency in the responses of pests and natural enemies to non‐crop habitats in the landscape. Local‐scale properties, such as the crop and non‐crop habitats immediately adjacent to the edge of focal fields, might support natural enemies with food and shelter and facilitate spillover into the fields during the cropping season (Anjum‐Zubair et al., 2010; Heimoana et al., 2017). However, adjacent habitats have also been reported to have negative effects, increasing abundance of pests rather than natural enemies (Zhao et al., 2013), or having no measurable influence (Fusser et al., 2017). Such inconsistency in the effects of local vegetation may result from the influence of landscape‐scale properties (Tscharntke et al., 2012). In the ‘intermediate landscape complexity hypothesis’, Tscharntke et al. (2012) predicted that provision of local‐scale resources to natural enemies is relatively ineffective in landscapes with extremely high or low levels of complexity because in the former case natural enemies already have abundant resources while in the latter resource availability is so poor that natural enemy communities are likely to be depauperate. In contrast, landscapes with moderate complexity and enough natural or semi‐natural non‐crop habitat to support an assemblage of natural enemies are considered to favor a response from the presence of resources at the local scale. While local‐scale effects can benefit from landscape‐scale heterogeneity (Chaplin‐Kramer & Kremen, 2012) this is not universally the case (Fusser et al., 2017).

The inconsistent effects of landscape diversity are also, in part, a reflection of the importance of considering multiple landscape properties of the habitats. To date, landscape composition (percentages of each habitat type in a given area) has been the most widely studied property. To find possible causes of the inconsistent effects, inclusion of other properties such as edge density (total boundary length of patches of each habitat type) can be advantageous. For example, Martin et al. (2019) found landscape composition as well as edge density of non‐crop habitats affected the strength of ecosystem services, including pest suppression. A third property is connectivity which considers the relative permeability of the landscape to a given taxon in terms of the suitability of each habitat type and the spatial positioning of these habitats; clearly something that might affect the movement of pest and natural enemy arthropods within an agricultural landscape (Blitzer et al., 2012; Rösch et al., 2013). In the absence of sufficient connectivity, even a high composition of favorable habitats may not facilitate the arrival of natural enemies in crop fields because of a lack of what amount to ‘stepping stone’ or ‘corridor’ features (Perović et al., 2010).

Here we integrate arthropod data from a survey of 24 Australian brassica vegetable fields, extending across four states (Western Australia, New South Wales, Victoria, South Australia, and Queensland) and 12 months, with information on adjacent habitats and multiple landscape properties (composition, edge density, and connectivity). Brassica vegetables, comprising familiar commodities, such as cabbage (*Brassica oleracea* var. *capitata*), cauliflower (*Brassica oleracea* var. *botrytis*), broccoli (*Brassica oleracea* var. *italica*), and Brussels sprouts (*Brassica oleracea* var. *gemmifera*), are one of the top 10 most widely grown crops in the world (Francisco et al., 2017). Global demand promotes intensive cultivation of these vegetables and facilitates their insect pests becoming commonplace in many agricultural landscapes. Brassicas are typically damaged by a complex group of insects including Lepidoptera and Hemiptera, among which the diamondback moth, and green peach aphid and cabbage are considered especially damaging. Consumer demand for unblemished and pest‐free produce contributes to high levels of pesticide use during production. Natural enemies such as spiders, predatory Coleoptera, and hymenopteran parasitoids attack brassica pest insects (Furlong et al., 2014) but the extent to which they provide adequate levels of biological control is usually constrained by pesticide use (Furlong et al., 2004) and a lack of knowledge of practical strategies for their enhancement by habitat management (Furlong et al., 2014; Gurr et al., 2018).

Improving prospects for use of biological control in brassica crops, as well as generating a broader understanding of the effects of differing spatial scales in arthropod dynamics are important goals. While it is intuitive that landscape properties potentially influence the effects of local properties on arthropods, the nature and strength of multi‐scale interactions for differing taxa and trophic levels are currently poorly understood. Accordingly, this study compiled data from a continent‐wide series of field surveys in Australia to investigate the within‐field effects of differing habitats in the areas immediately adjacent to the crop together with impacts of landscape properties to the scales of up to five kilometers from focal fields. Specifically, we tested three hypotheses. First, that the nature of habitat in areas immediately adjacent to the crop leads to detectable differences in the numbers of arthropods in different areas of the crop field. For this we developed a novel index that quantified the differences between insect counts in the field centers (taken as a baseline) and counts of the same taxon in the edge of the crop adjacent to each type of adjacent habitat. Second, that the wider scale landscape composition, edge density, and connectivity affect arthropod abundance in crop fields. To test this, we used insect counts only from the center of fields so that these response data would be minimally affected by edge effects caused by adjacent habitats. Third, that landscape composition, edge density, and connectivity affect the strength of the short‐range effects mediated by the habitat immediately adjacent to the crop. We tested this by using the edge effects measured in the first hypothesis as response data.

## 2 MATERIALS AND METHODS

### 2.1 Study sites

**FIGURE 1:**
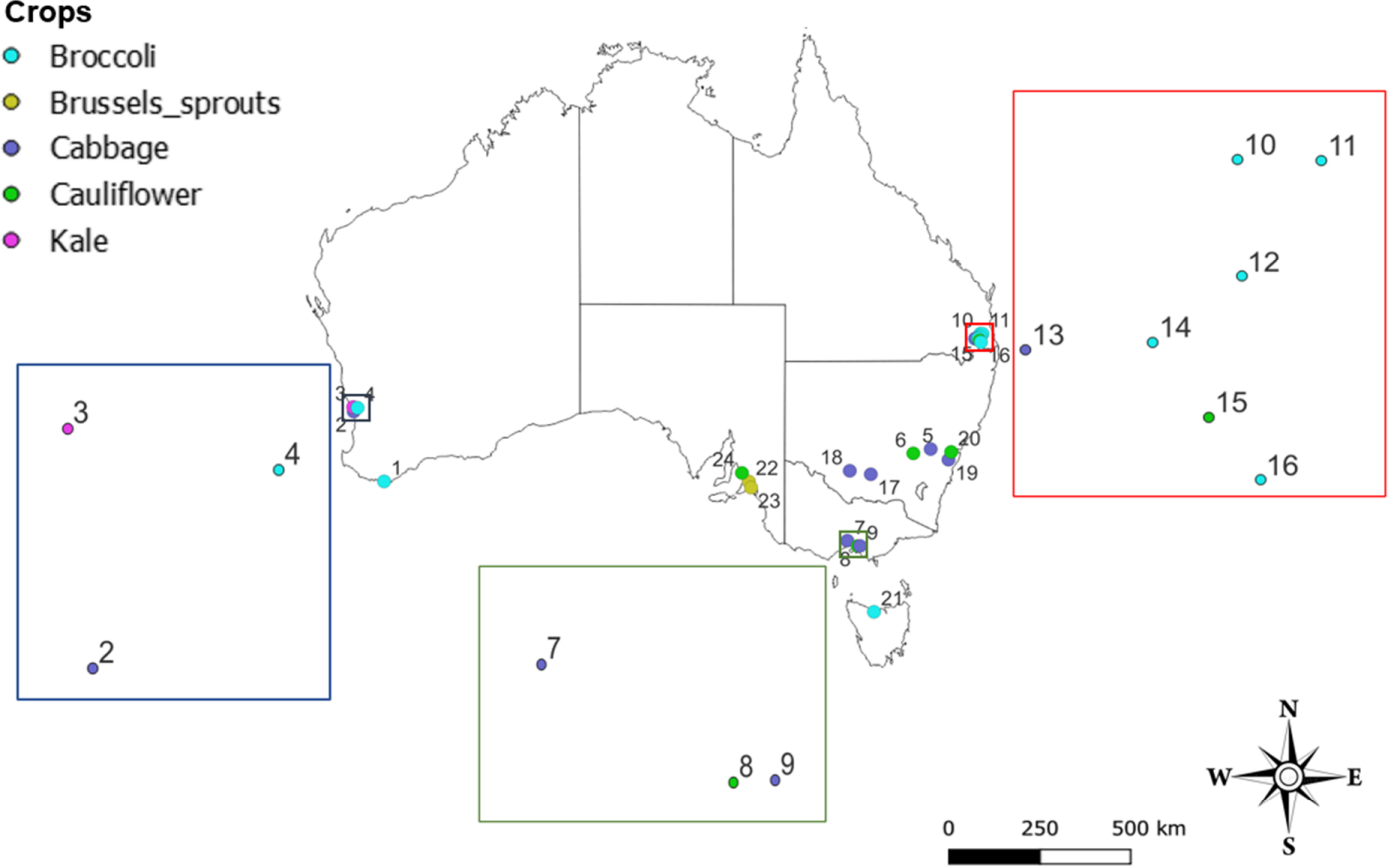
Locations of data collection sites. Numbers indicating the serial of the locations are listed in Table S1. The map was created Arc map in ArcGIS 10.6.

Sites were located across the 15 regions (Great Southern, Mid‐west, Peel, Central West, Cranbourne, Greater Malborne, Dalmore, Lockyer Valley, Mount Sylvia, Mulgowie, Rivenia, Camden, Richmond, Forth, Langhorne Creek) of the southern half of Australia, representing the main brassica vegetable production regions (Figure 1; Table S1). Data were collected from one focal field on each of 24 farms, each located at least 10 km from other surveyed farms to ensure spatial independence (Thiele & Markussen, 2012). Fields with a minimum area of 20 × 20 m^2^ were sampled. Sampling was carried out at least 3 days after insecticide application.

### 2.2 Focal crops

The brassica vegetables (five species) grown in the focal fields were cabbage (eight fields), cauliflower (five fields), broccoli (eight fields), Brussels sprouts (two fields), and kale (one field).

### 2.3 Arthropod sampling

Between February 2018 and January 2019 (Summer 2017–2018, Winter 2018, and Summer 2018–2019), arthropod abundance was assessed in the central zone of each focal field and 2 m into the field from each edge bordered by a different type of adjacent habitat. The different habitats bordering a focal field ranged from two to four different types (see ‘Section 2.4’ below). The geographical dispersion of the sites, distance from the home campus (Orange, New South Wales), and labor availability necessitated the use of a rapid assessment protocol for arthropods. For each sampling zone, 10 plants were randomly selected, and a visual search was carried out for arthropods. Counts were of all arthropods detected on plants at the juveniles of lepidopterans, juveniles, and adults of coleopterans and hemipterans. Eggs were not counted. All surveys were non‐destructive in nature and conducted on dry days between 10.00 and 16.00 h. All sides of leaves, stems, flowers, and fruits of each plant were hand‐searched for the presence of arthropods (pests and natural enemies). Arthropods were identified in situ to appropriate taxonomic levels and recorded as point count data. Each of the 24 fields was sampled once only.

### 2.4 Adjacent habitats

The land uses and vegetation adjacent to each edge of each field were visually identified and recorded. These were classified into five categories: brassica cropland, cropland of other species (non‐brassica crops), woodland or woody vegetation (trees, shelterbelts, riparian regions, and shrubs), pastures (usually dominated by cultivated or uncultivated grasses but occasionally of lucerne (*Medicago sativa*)), and water bodies.

### 2.5 Landscape‐scale properties

Landscape properties were extracted from the Dynamic Land Cover Dataset (DLCD) sourced from Lymburner et al. (2010) using ArcGIS 10.6 (ESRI, 2017). Data on three landscape properties; landscape composition, edge density, and connectivity were assessed at the spatial scales of 250, 500, 1000, 2500, and 5000 m radii from the field center. Landscape composition or percentage of different habitats within a spatial scale was measured using the spatially specific proportional area approach (Schmidt et al., 2008). Habitats were classified in the same categories as the adjacent land‐use analysis except that it was not possible to partition cropland into brassica and non‐brassica crops. Edge density of each land use was calculated according to Martin et al. (2019) and is defined as the total length of one land‐use type divided by the total landscape area of a given spatial scale. The unit of edge density was meters per hectare. A ‘cost‐distance’ analysis was utilized to measure landscape connectivity of habitats that determine the movement of an arthropod from sources within the selected spatial scales to the focal crop field (Perović et al., 2010). Ten sets of cost‐ratios (Table 1) were tested following the cost‐ratios used in earlier studies by Perović et al. (2010) and Chardon et al. (2003) using the ‘cost‐distance’ tool in ArcGIS 10.6. The values in each of these ratios represented the hypothetical costs to a given arthropod taxon of moving through each type of habitat. Land‐use with a high cost is less favorable for movement of a given type of arthropod. Thus, that land‐use is a barrier or indicates less connectivity with the focal crop field to facilitate the arrival of the arthropod in the focal field. Conversely, the low‐cost land‐uses are highly favorable and indicate well connectivity with the focal field. Two metrics of ‘cost‐distance’ analysis were used for further statistical analysis. The summation of costs of all cells in the cost raster (digitized aerial image layer) of a spatial scale represents the ‘cost‐area’ metric (Perović et al., 2010). The cost‐area for each spatial scale indicates the overall connectivity of that scale. The second metric was cost‐path, representing the lowest cumulative cost to reach the crop field (destination) from sources within a spatial scale (Perović et al., 2010).

**TABLE 1:** Assigned cost‐ratios used in the cost‐distance analysis. Lowest cost (1) indicates highly favorable habitats and highest cost (100) indicates highly unfavorable habitats for a taxon to arrive a focal field from a source in the landscape

Land‐use types	Cost‐ratios									
	r1	r2	r3	r4	r5	r6	r7	r8	r9	r10
Water bodies	4	4	20	20	20	1	1	1	100	100
Croplands	2	4	10	20	10	1	1	1	1	100
Pastures	1	1	2	2	1	2	2	100	10	4
Woodland	1	1	1	1	1	4	2	20	20	1

### 2.6 Statistical analyses

1. Edge effects: The effects of adjacent habitats on in‐field abundance of arthropods were measured by comparing the arthropod abundance in the crop edge nearest each type of adjacent habitat with equivalent abundance at the center of the field (that is, edge minus center). Before the comparison, the observed arthropod abundances on 10 plants were pooled to minimize the number of zeros (Baillod et al., 2017) and then standardized by subtracting the mean and dividing by the standard deviation of the sample (i.e., z‐score). For each field, the comparison between edge and center of the field generated an ‘edge effect’. Negative values of ‘edge effect’ indicated a suppressive effect of the adjacent habitat whereas positive values indicated that the adjacent habitat promoted arthropod abundance. The statistical significance of each positional effect was tested using one‐sample *t‐*tests (de Araújo & do Espírito‐Santo Filho, 2012). This analysis was conducted for each type of adjacent habitat and arthropod taxon to test the null hypothesis that arthropod abundances were consistent between field centers and edges.
2. Effects of landscape properties on the abundance of arthropods at field center: To assess the effects of landscape properties with the minimum possible influence of adjacent habitats, data for each arthropod taxon collected from only the field centers (response variables) were regressed against landscape properties (predictor variables) as fixed factors in a linear mixed model (LMM). The model included crop growing seasons of sampling, crop species, and regions of sites as random factors and was run in R‐studio version 4.0.3. A Gaussian distribution with ‘identity’ link‐function was used in the model: Response variable ~ Predictor variables + (1|Cultivated crop) + (1|Region of the site) + (1|Sampling season). Link‐functions were untied, before creating the graphs, using the following command: predict(model, type = “response”). The global model included all of the predictors of landscape properties (composition, edge density, cost‐area, and cost‐path) at the five spatial scales for each taxon. Due to the presence of multicollinearity, the predictors with VIF < 5 (Tables S2, S3) were excluded from the final model. This regression analysis investigated whether landscape properties significantly affect the abundance of pests and natural enemies of brassica vegetables at the center of the focal fields.
3. Effects of landscape properties on the edge effects: This analysis tested the hypothesis that the strength of the ‘edge effects’ of field adjacent habitats on the abundance of arthropods in the crop edges is affected by landscape‐scale properties. Significant edge effects of the adjacent habitats on arthropod abundance (response variable) found in the ‘Statistical analytics’ Section 1 were regressed against all the predictor variables of landscape composition, edge density, and connectivity at the five spatial scales. Variables with multicollinearity (VIF > 5) were handled similarly as described previously for the analysis of arthropod abundance at the field center. Linear mixed models (LMM) with a Gaussian error distribution (‘identity’ link‐function) and crop growing seasons of sampling, crop species, and regions of sites as random factors were run to regress the edge effects (response variables) against the best variable from each landscape property (predictors). Graphs were created after untied the link as described in the previous section. The structure of the models was similar as described in the previous section (Effects of landscape properties on the abundance of arthropods at field center).

## 3 RESULTS

### 3.1 Arthropod abundance

A total of 811 pests and 355 natural enemies were recorded from the 24 brassica vegetable fields (Table S5). Major pest taxa were diamondback moth (285), whiteflies (Aleyrodidae) (111), green peach aphid (212), and cabbage aphid (89). Numerically dominant natural enemy taxa were ladybirds (119) and spiders (73).

### 3.2 Edge effects of adjacent habitats

**FIGURE 2:**
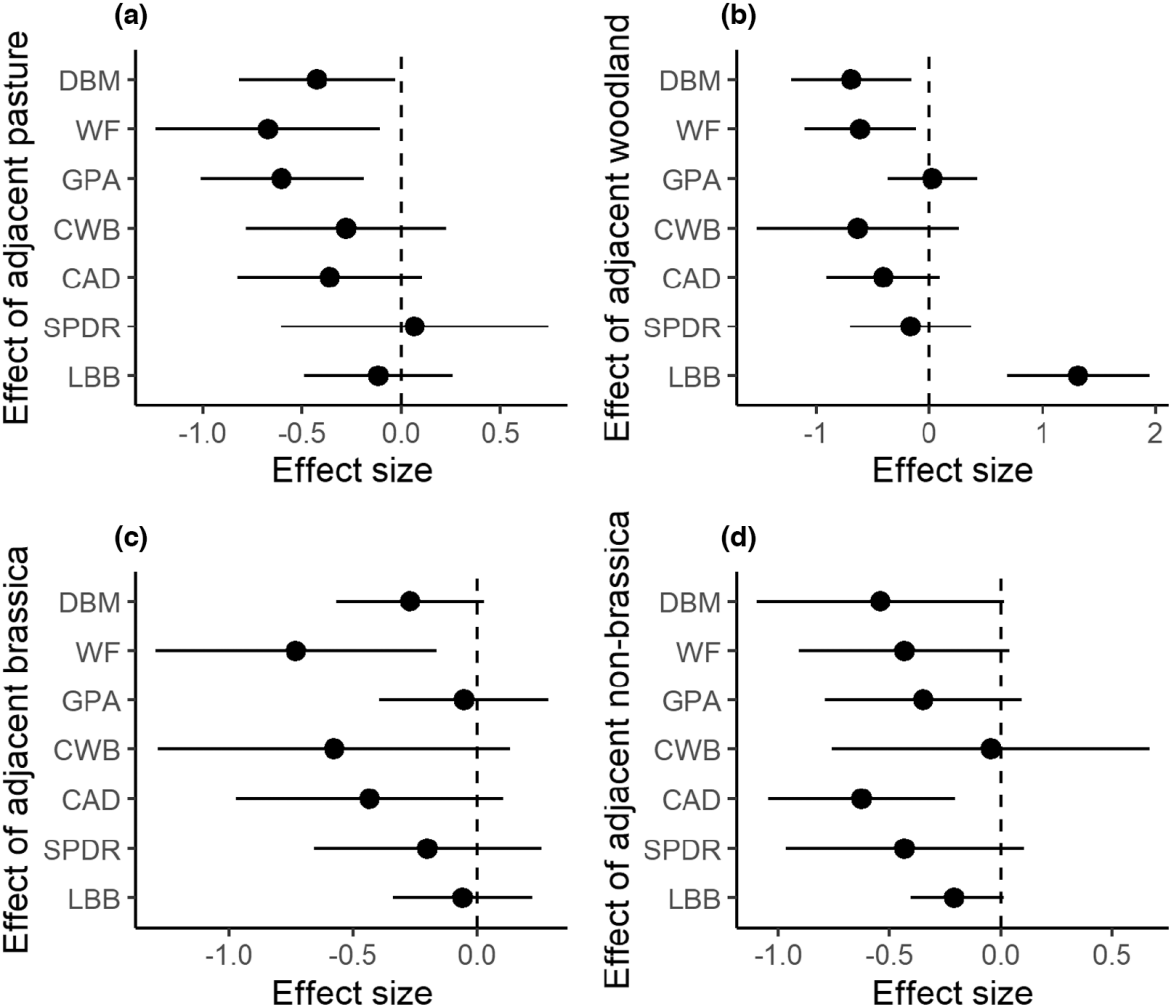
Edge effects of adjacent habitats (a) pasture, (b) woodland, (c) brassica crops, and (d) non‐brassica crops on the distribution of arthropods in brassica fields (mean differences between field center and field edges with 95% confidence interval). Negative values indicate adjacent habitats that decrease the abundances of arthropods in the nearby edge compared with the field center while positive indicate an increase. DBM = diamondback moth (*n* = 676 for pasture edge, *n* = 187 for woody vegetation edge, *n* = 1296 for brassica crop edge, *n* = 333 for non‐brassica crop edge), WF = whiteflies (*n* = 169 for pasture edge, *n* = 208 for woody vegetation edge, *n* = 180 for brassica crop edge, *n* = 225 for non‐brassica crop edge), GPA = green peach aphid (*n* = 156 for pasture edge, *n* = 649 for woody vegetation edge, *n* = 954 for brassica crop edge, *n* = 279 for non‐brassica crop edge), CWB = cabbage white butterfly (*n* = 13 for pasture edge, *n* = 33 for woody vegetation edge, *n* = 72 for brassica crop edge, *n* = 0 for non‐brassica crop edge), CAD = cabbage aphid (*n* = 234 for pasture edge, *n* = 176 for woody vegetation edge, *n* = 270 for brassica crop edge, *n* = 63 for non‐brassica crop edge), SPDR = predatory spiders (*n* = 273 for pasture edge, *n* = 154 for woody vegetation edge, *n* = 134 for brassica crop edge, *n* = 54 for non‐brassica crop edge), and LBB = predatory ladybirds (*n* = 104 for pasture edge, *n* = 913 for woody vegetation edge, *n* = 198 for brassica crop edge, *n* = 27 for non‐brassica crop edge).

There were robust effects of adjacent habitats, despite aggregation of data from fields dispersed over a wide geographical area and sampling that extended across multiple seasons of the year as well as differing species of brassica crop vegetables, differing local conditions (such as precise pesticidal regime and date from last application), field sizes, and so on. Presence of pasture adjacent to brassica fields was associated with lower abundance of the major pests, diamondback moth, whiteflies, and green peach aphid in the edge compared to the field center (*p*‐values = .035, .022 and .006, respectively) (Figure 2a). Similarly, woodland reduced diamondback moth (*p*‐value = .013) and whiteflies (*p*‐value = .017) but had the opposite effect on ladybirds, increasing their abundance in the adjacent crop (*p*‐value <.001) (Figure 2b). The presence of an adjacent brassica crop was significantly associated with a lower abundance of whiteflies in the focal brassica crop (*p*‐value = .014) (Figure 2c). Presence of non‐brassica crops reduced abundance of cabbage aphid (*p*‐value = .005) (Figure 2d).

### 3.3 Effects of landscape properties on field center abundance of arthropods

**FIGURE 3:**
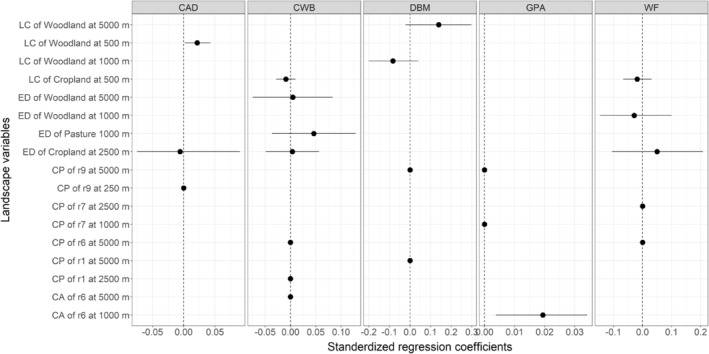
Effects of landscape variables on the abundance of pests in the field center. CAD, cabbage aphid; CWB, cabbage white butterfly; DBM, diamondback moth diamondback moth; GPA, green peach aphid; WF, whiteflies. CP, cost path of landscape connectivity; CA, cost area of landscape connectivity; ED, edge density; LC, landscape composition.

The presence of woody vegetation within 500 m of the focal field increased abundance of cabbage aphid in the center of focal fields (Figure 3; Table S2). For the other major aphid pest species, green peach aphid, the cost‐area metric of landscape connectivity was significantly associated with increased abundance in the centers of focal fields. The cost‐ratio r6, that assigned croplands as the most favorable land‐use, pastures half as favorable and woody vegetation half again (Table 1), applied at a landscape radius of 1000 m, best explained in‐field abundances of this pest (Figure 3; Table S2). No taxa were significantly affected by the cost‐path metric for the measurement of landscape connectivity, or by the edge densities of any land‐uses at any spatial scale.

**FIGURE 4:**
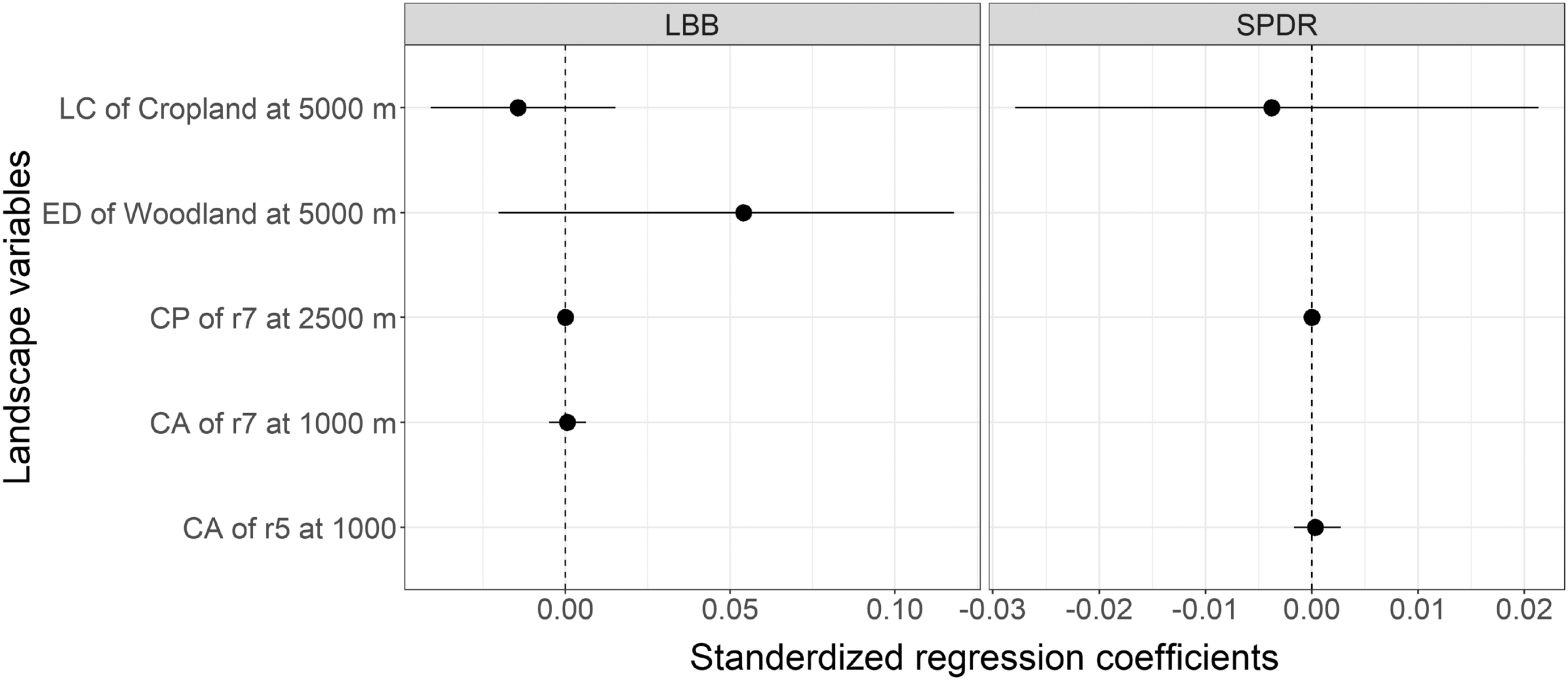
Effects of landscape variables on the abundance of natural enemies in the field center. LBB, predatory ladybirds; SPDR, predatory spiders. ED, edge density; CP, cost path of landscape connectivity; CA, cost area of landscape connectivity; LC, landscape composition.

Natural enemy abundance in the field center was not influenced significantly by any of the landscape variables (Figure 4; Table S2).

### 3.4 Effect of landscape properties on the edge effects of adjacent habitats

**FIGURE 5:**
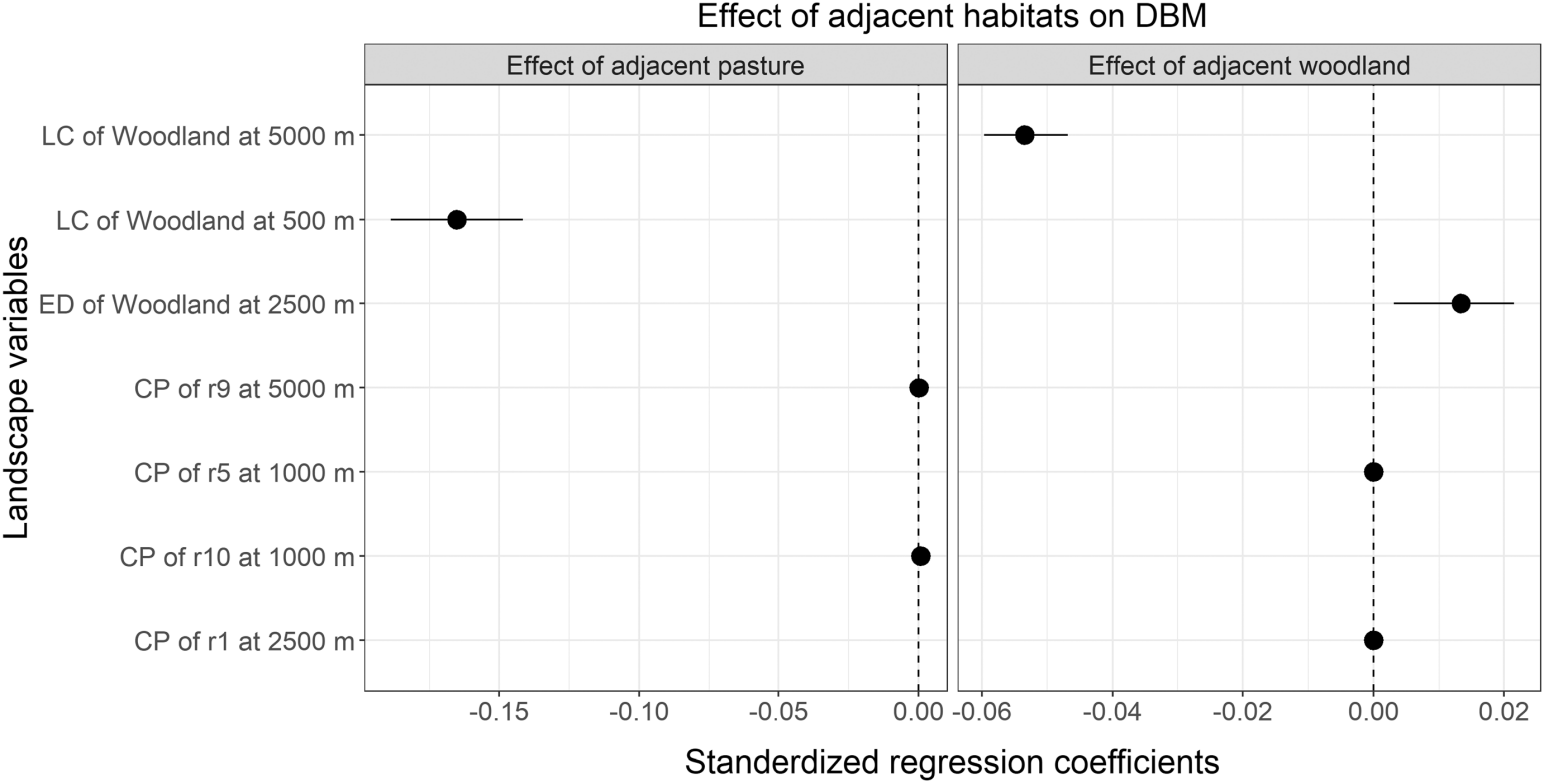
Effect of landscape variables on the edge effect of field adjacent habitats on DBM, diamondback moth diamondback moth; CP, cost path of landscape connectivity; ED, edge density; LC, landscape composition.

For diamondback moth, there were significant effects of landscape‐scale properties on the edge effects caused by adjacent pasture. Abundance of woodland within 500 m of focal fields significantly strengthened the effect of adjacent pasture on reducing abundance of diamondback moth (Figure 5; Table S3). A significant strengthening of the effect of adjacent woodland on reduced abundance of diamondback moth was also observed in the negative association of adjacent woodland and the landscape composition of woodland at the 5000 m scale (Figure 5; Table S3). However, edge density of woodland at 2500 m weakened the effect of woodland on diamondback moth.

**FIGURE 6:**
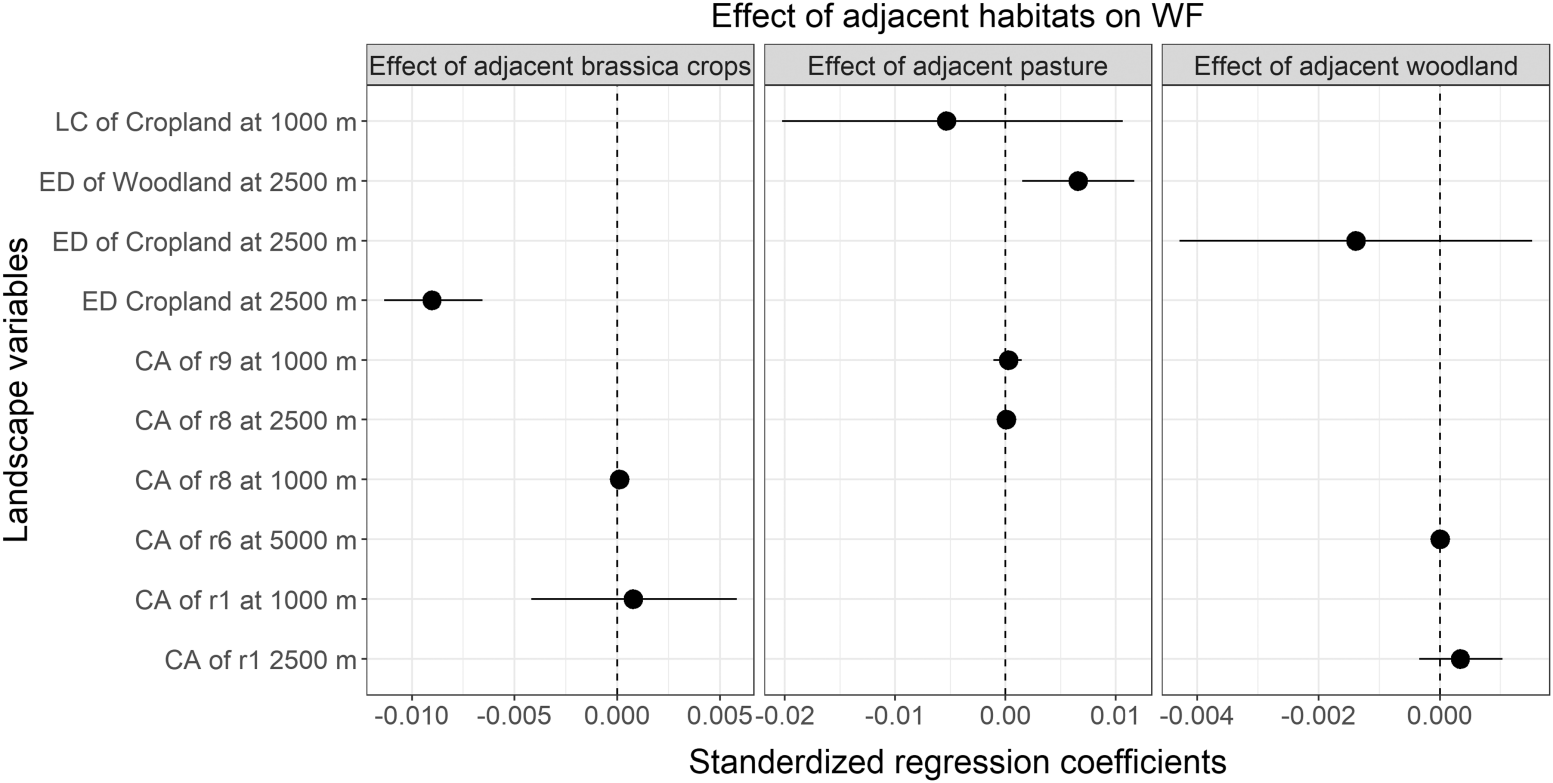
Effect of landscape variables on the edge effect of field adjacent habitats on WF, whiteflies. CA, cost area of landscape connectivity; ED, edge density; LC, landscape composition.

For whiteflies, edge density of habitats in the landscape, though not significant in isolation, significantly influenced local edge effects. Cropland edge density at 2500 m scale strengthened the edge effect of adjacent brassica crops on the reduced abundance of the pest. However, edge density of woodland at the same scale diminished the suppressive effect on abundance of whiteflies associated with the presence of field adjacent pasture. The strength of the effect of adjacent woodland on whiteflies was not significantly influenced by any of the landscape variables (Figure 6; Table S3).

**FIGURE 7:**
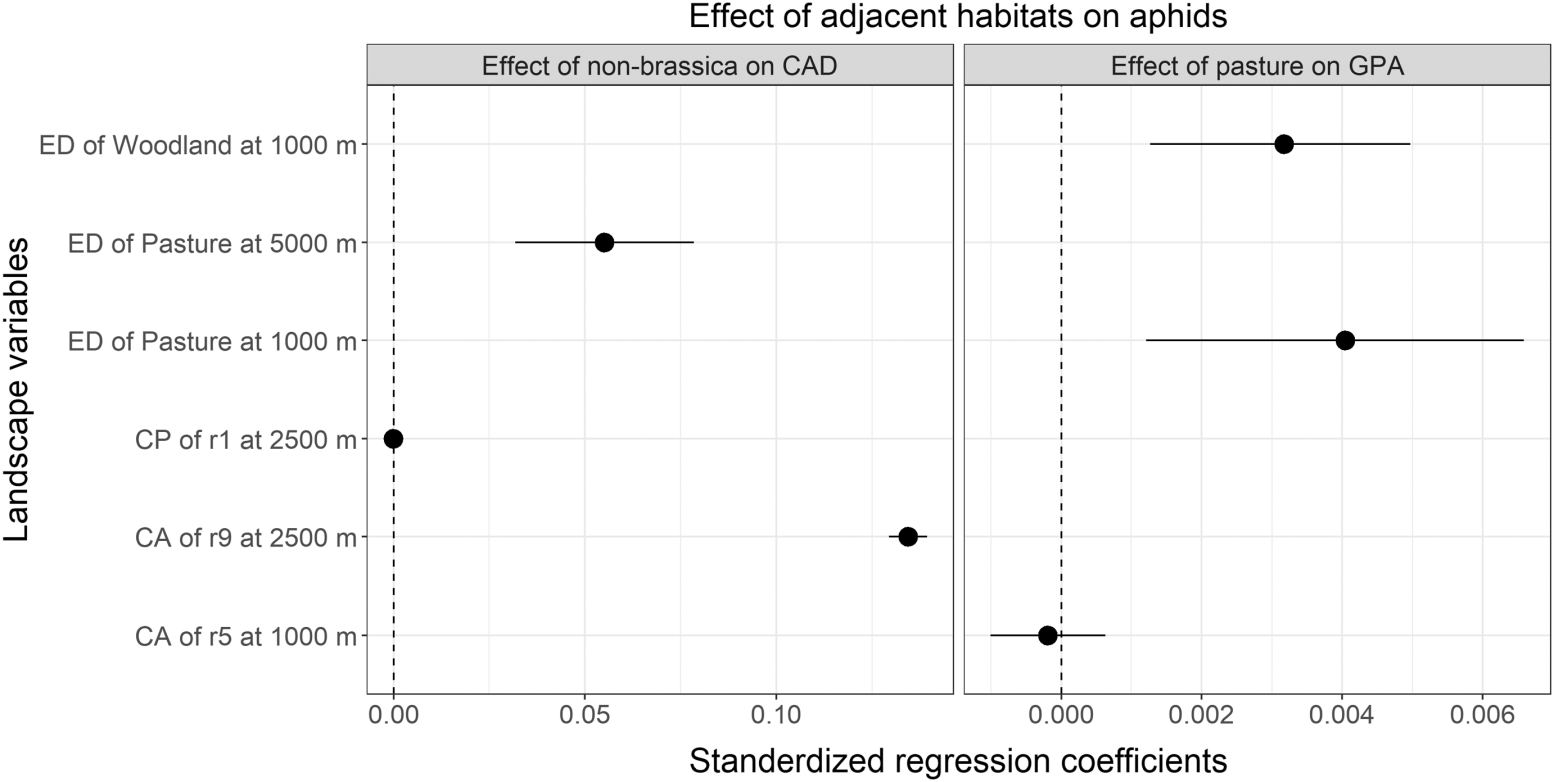
Effect of landscape variables on the edge effect of field adjacent habitats on aphids CAD, cabbage aphid and green peach aphid. CP, cost path of landscape connectivity; CA, cost area of landscape connectivity; ED, edge density.

For cabbage aphid, weakening of the edge effects caused by adjacent non‐brassica crops was observed in the association with the edge density of pastures at 5000 m scale and cost‐area metric for the cost‐ratio r9 at the scale of 2500 m (Figure 7; Table S3). For r9, woodland, pastures, and water bodies were assigned to be 20, 10, and 100‐fold less favorable than croplands, respectively. For the second aphid species, green peach aphid, *e*dge density of woodland and pasture at the 1000 m scale diluted the suppressive effect of adjacent pasture on abundance (Figure 7; Table S3).

**FIGURE 8:**
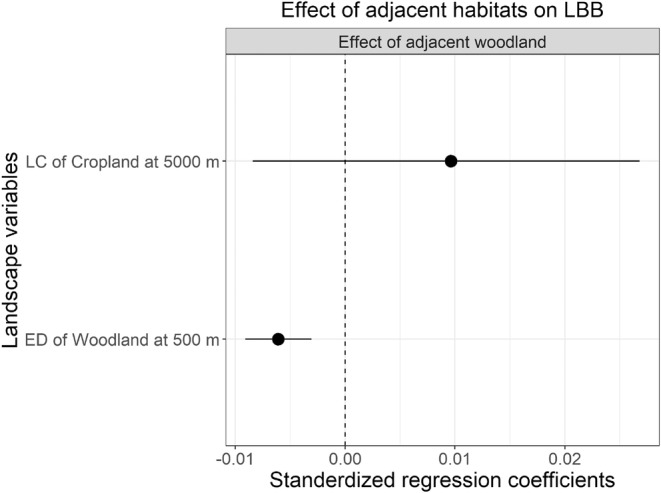
Effect of landscape variables on the edge effect of field adjacent habitats on LBB, predatory ladybirds. ED, edge density; LC, landscape composition.

For ladybirds, woodland edge density at the 500 m scale diluted the enhancing effect of adjacent woodland (Figure 8; Table S3).

## 4 DISCUSSION

This study demonstrates multiple, strong effects of immediately adjacent habitats on the arthropods in brassica crops. We recorded significant negative effects on the abundance of all major pest taxa in the crop edges adjacent to at least one type of adjacent land‐use (such as pasture or woodland) compared with the center of the crop. The only natural enemy taxon to respond positively was ladybirds (Coccinellidae) which were more numerous in crop edges adjacent to woodland, the habitat with less fewer diamondback moth and whitefly. Landscape‐scale properties, in contrast, had few distinct effects on in‐crop densities of arthropods; only the two aphid species (*B. brassicae* and *M. persicae*) significantly affected. Notably, however, there were many instances of landscape properties affecting the strength of the effects caused by adjacent habitats, highlighting the importance of this multi‐scale study.

### 4.1 Edge effects

Field‐adjacent pasture, woodland, brassica, and non‐brassica crops all reduced the abundance of specialist brassica pests in the adjacent brassica crop edge compared to the field center. This effect was evident across pest families and feeding guilds: diamondback moth (lepidopteran chewing feeders), and whiteflies and cabbage aphid (both hemipteran sucking feeders). This effect is consistent with the resource concentration hypothesis (Root, 1973) whereby the establishment and persistence of specialist herbivores on their host plant may be disrupted by the presence of nearby non‐host plants and operates separately to any effect mediated by natural enemies. Resource concentration effects can operate by several mechanisms including the non‐host plants serving as a barrier to the arrival of and subsequent damage by specialist herbivores on host plants. This is most conspicuously possible in the observed effects of woodland; large and perennial plants that would potentially block visual and olfactory cues as well as physical movement of arthropods (Gurr et al., 2018). Associated with this, and likely to have been important across multiple types of non‐crop habitats, is the fact that these habitats will not have supported populations of the pest (Liu et al., 2005). Accordingly, spillover of pests from these patches is likely to be negligible. Whiteflies also exhibited reduced abundance in the crop edges adjacent to other brassica crops, woody and pasture vegetation, but the in‐field assessment protocol used in this study did not allow discrimination of the specialist whitefly *Aleyrodes proletella*, and more polyphagous species such as *Trialeurodes vaporariorum* and *Bemisia tabaci*. Accordingly, it is not possible to unambiguously attribute the effects of woody and pasture vegetation to resource concentration effects because non‐brassica specialist or generalist herbivores may have been hosted by some plant species in the non‐crop vegetation (McKenzie et al., 2004) from which spillover may have subsequently occurred. However, the effect of adjacent brassica crops on whiteflies reflects a resource dilution effect which was evident in the findings of Otway et al. (2005), where there was a lower abundance of specialist insect herbivores due to the richness of their favorable habitats near the focal crops.

Notwithstanding the operation of resource concentration effects, natural enemy effects could also have operated at this local scale because ladybirds, a taxon of predators that attacks all brassica pests (Lira et al., 2019), were significantly more numerous in brassica crop edges adjacent to woodland. This effect may have occurred if the woodland provided resources, such as pollen, prey, and shelter, that led to a high density of ladybirds and so constituted donor habitat from which these predators colonized nearby crop fields when prey became available (Heimoana et al., 2017; Thomson & Hoffmann, 2013). As the effect of ladybirds on reduced abundance of the brassica pests was not measured in this study, the present evidence for a natural enemy effect is limited to correlational relationships and broader evidence from the foregoing references of the role of ladybirds as predators in brassica crop systems.

### 4.2 Effects of landscape properties on field center abundance of arthropods

Landscape compositions with high proportions of woody vegetation at the 500 m radius scale were associated with increased abundance of cabbage aphid in field centers. In broad agreement, the numbers of the other aphid species, green peach aphid were increased by high‐cost area values from the landscape connectivity analysis. In that analysis, the relative non‐favorability assigned to woodland was four‐fold greater than the favorability of crops, and double that assigned to pastures. Thus, landscapes with high cost had relatively abundant woodland. This apparent promotion of aphids in crops by aspects of woodland predominance in the landscape is contrary to the often held belief that non‐crop vegetation in the landscape leads to herbivore suppression, potentially through enhanced natural enemy activity (Bianchi et al., 2006). However, that general notion has been eroded by the recent meta‐analysis of Karp et al. (2018) and those authors suggested that pest suppression was not axiomatic with non‐crop vegetation. In this present study, the higher in‐field abundance of cabbage aphid in fields set in woodland‐rich landscapes could have resulted from natural enemies tending to remain in non‐crop areas rather than in crops in order to exploit the availability of shelter and a diversity of food resources provided by the relative structural complexity and botanical diversity of woody plant communities (Tscharntke et al., 2016). While this effect is counter to the short‐range effect observed in the present study, as woodland immediately adjacent to crop fields promoted ladybirds at field edges, scale effects may account for this (Bhar & Fahrig, 1998). Woodland adjacent to crops would allow ladybirds to diurnally move to crops to forage for prey yet return to woodland for shelter from environmental extremes. Such movement by ladybirds from crop field to adjacent shelterbelt was observed in a study in wheat (Dong et al., 2015).

### 4.3 Effect of landscape properties on the edge effects of adjacent habitats

Testing for influence of landscape properties on the edge effects that were calculated by comparing arthropod abundances in field centers with field edges detected significant impact for several pests and one natural enemy taxon. For the specialist herbivore of brassica vegetables, diamondback moth, the lower abundance associated with adjacent pastures and woodland was enhanced by the presence of woodland habitat in the wider composition of the landscape. That is, a landscape dominated by habitats that were strongly unfavorable to this specialist herbivore (Hu et al., 1997) exaggerated differences in in‐field abundances of this pest, with much lower abundance in the edges close to pastures and woodland.

Higher abundance of woodland in terms of edge density and landscape connectivity was found to dilute several edge effects of adjacent habitats on pest suppression (adjacent woodland on diamondback moth, adjacent pasture on whiteflies and green peach aphid, adjacent non‐brassica crops on cabbage aphid) and enhancing natural enemy abundance (adjacent woodland on predatory ladybirds). The increasing edge density and connectivity of woodland indicated increasing landscape complexity which is consistent with the intermediate landscape complexity hypothesis, whereby the strength of local‐scale factors predicted to be weakened in complex landscapes (Tscharntke et al., 2012).

## 5 CONCLUSIONS

The key finding of this study was the existence of strong influences of landscape properties on the effect of local habitats that can shape the in‐field abundance of pest and natural enemy arthropods. Initially, comparing the abundances of arthropods in field edges with the field center, suppressive effects of diverse adjacent habitats on several key pests were detected. The enhanced abundance of a generalist natural enemy taxon (ladybirds) promoted by woodland adjacent to focal fields was consistent with a contribution of trophic top‐down effects in reducing the pest abundance. Secondly, assessment of effects of landscape properties at the field center revealed significant effects for only two pest taxa. Finally, the impact of landscape properties on edge effects of adjacent habitats demonstrated effects on pests and ladybirds.

Our findings demonstrate that disregarding the effects of landscape‐scale drivers such as composition, edge density, and connectivity can result in poor capacity to understand the responses of arthropods to more local properties such as immediately adjacent habitat. While these results are of use to identify fields in settings with high pest risk and low levels of biological control and suggest strategies that might reduce pest risk (e.g., establishment or restoration of field margin woody vegetation), more studies combining local and landscape drivers are needed to fully realize the potential for non‐chemical pest suppression.

## AUTHOR CONTRIBUTIONS

**Salma Akter:** Formal analysis (lead); writing – original draft (lead). **Syed Z. M. Rizvi:** Resources (equal). **Ahsanul Haque:** Resources (equal). **Olivia L. Reynolds:** Supervision (supporting); writing – review and editing (equal). **Michael Furlong:** Writing – review and editing (equal). **Maria C. Melo:** Resources (equal). **Terry Osborne:** Resources (equal). **Jianhua Mo:** Resources (equal); writing – review and editing (equal). **Simon McDonald:** Formal analysis (equal); writing – review and editing (equal). **Anne C. Johnson:** Writing – review and editing (equal). **Geoff Gurr:** Conceptualization (lead); supervision (lead); writing – review and editing (lead).

## Supporting information


Dataset S1.

## Data Availability

All data that support the findings of this study will be openly available at the Dryad Digital Repository.
