# Supplementary material for: Continent‐wide evidence that landscape context can mediate the effects of local habitats on in‐field abundance of pests and natural enemies
Source: Ecol Evol. 2023 Jan 11;13(1):e9737. doi: 10.1002/ece3.9737 (PMC9833983; doi:10.1002/ece3.9737)
Supplement: Supplementary file 1 — Dataset S1. [file ECE3-13-e9737-s001.docx]

Table S1. Location information of the sites

| Field ID | Locations | Regions | States | Latitude | Longitude | Cultivated crops | Seasons of sampling |
| --- | --- | --- | --- | --- | --- | --- | --- |
| 1 | Albany | Great southern | Western Australia | -35.0514 | 117.4824 | Broccoli | Summer 2017-18 |
| 2 | Gingin | Mid west | Western Australia | -31.5685 | 115.7025 | Cabbage | Summer 2017-18 |
| 3 | Gingin | Peel | Western Australia | -31.3343 | 115.6715 | Kale | Summer 2018-19 |
| 4 | Gingin | Mid west | Western Australia | -31.3744 | 115.9271 | Broccoli | Summer 2017-18 |
| 5 | Kelso | Central west | New South Wales | -33.4542 | 149.5921 | Cabbage | Summer 2017-18 |
| 6 | Canowindra | Central west | New South Wales | -33.6681 | 148.5743 | Cauliflower | Summer 2017-18 |
| 7 | Werribee | Cranebourne | Victoria | -37.9309 | 144.7088 | Cabbage | Winter 2018 |
| 8 | Devon Meadows | Greater Melborne | Victoria | -38.1818 | 145.3208 | Cauliflower | Summer 2018-19 |
| 9 | Dalmore | Dalmore | Victoria | -38.1770 | 145.4527 | Cabbage | Winter 2018 |
| 10 | Lockyer Valley | Lockyer vally | Queensland | -27.5236 | 152.5016 | Broccoli | Winter 2018 |
| 11 | Lockyer Valley | Lockyer vally | Queensland | -27.5243 | 152.6263 | Broccoli | Summer 2018-19 |
| 12 | Forest Hill | Lockyer vally | Queensland | -27.6858 | 152.5068 | Broccoli | Winter 2018 |
| 13 | Lockyer Valley | Mt Sylvia | Queensland | -27.7869 | 152.1831 | Cabbage | Winter 2018 |
| 14 | Mount Sylvia | Mt Sylvia | Queensland | -27.7770 | 152.3743 | Broccoli | Summer 2017-18 |
| 15 | Mount Sylvia | Mt Sylvia | Queensland | -27.8806 | 152.4579 | Cauliflower | Winter 2018 |
| 16 | Mulgowie | Mulgowie | Queensland | -27.9670 | 152.5349 | Broccoli | Winter 2018 |
| 17 | Riverina | Rivenia | New South Wales | -34.7010 | 146.0853 | Cabbage | Summer 2017-18 |
| 18 | Riverina | Rivenia | New South Wales | -34.5304 | 144.8531 | Cabbage | Summer 2017-18 |
| 19 | Camden | Camden | New South Wales | -33.9849 | 150.6366 | Cabbage | Summer 2017-18 |
| 20 | Richmond | Richmond | New South Wales | -33.5981 | 150.7940 | Cauliflower | Summer 2017-18 |
| 21 | Forth | Forth | Tasmania | -41.2076 | 146.2708 | Broccoli | Summer 2018-19 |
| 22 | Mount Barker | Langhorne_Creek_SA | South Australia | -35.0593 | 138.8965 | Brussels_sprouts | Summer 2017-18 |
| 23 | Langhorne Creek | Langhorne_Creek_SA | South Australia | -35.3527 | 139.0273 | Brussels_sprouts | Summer 2017-18 |
| 24 | Port Gawler | Langhorne_Creek_SA | South Australia | -34.6361 | 138.4898 | Cauliflower | Summer 2018-19 |

Table S2. Landscape properties at different spatial scales influenced the arthropod abundance at the field centre.

| **Insect_taxa** | **Landscape_variables** | **Lower CI** | **Upper CI** | **Standerdized Coefficients** | **VIF** | **t value** |
| --- | --- | --- | --- | --- | --- | --- |
| DBM | LC of Woodland at 5000 m | -2.19E-02 | 2.98E-01 | 1.40E-01 | 2.521345 | 1.592 |
|  | LC of Woodland at 1000 m | -2.01E-01 | 4.05E-02 | -8.33E-02 | 2.463814 | -1.319 |
|  | CP of r9 at 5000 m | -4.44E-06 | 2.54E-06 | -9.57E-07 | 1.235124 | -0.506 |
| CWB | CP of r1 at 2500 m | -1.89E-05 | 5.48E-06 | -6.71E-06 | 1.223276 | -0.918 |
|  | CP of r6 at 5000 m | -1.10E-06 | 9.64E-06 | 4.27E-06 | 1.203189 | 1.325 |
|  | CA of r6 at 5000 m | -1.28E-04 | 9.67E-05 | -1.51E-05 | 1.090722 | -0.225 |
|  | ED of Pasture 1000 m | -3.67E-02 | 1.29E-01 | 4.61E-02 | 1.326557 | 0.93 |
|  | ED of Woodland at 5000 m | -7.43E-02 | 8.37E-02 | 4.69E-03 | 1.779414 | 0.099 |
|  | ED of Cropland at 2500 m | -4.91E-02 | 5.65E-02 | 3.71E-03 | 1.387108 | 0.117 |
|  | LC of Cropland at 500 m | -2.81E-02 | 1.00E-02 | -9.03E-03 | 1.299231 | -0.79 |
| WF | ED of Cropland at 2500 m | -1.05E-01 | 2.08E-01 | 5.00E-02 | 1.671447 | 0.635 |
|  | CP of r7 at 2500 m | -4.05E-05 | 2.30E-05 | -8.29E-06 | 1.316081 | -0.487 |
|  | CP of r6 at 5000 m | -1.67E-05 | 7.91E-06 | -4.40E-06 | 1.067297 | -0.692 |
|  | ED of Woodland at 1000 m | -1.46E-01 | 9.98E-02 | -2.88E-02 | 1.73269 | -0.438 |
|  | LC of Cropland at 500 m | -6.62E-02 | 3.10E-02 | -1.84E-02 | 1.48248 | -0.664 |
| CAD | LC of Woodland at 500 m | 1.56E-03 | 4.28E-02 | 2.16E-02 | 1.034677 | 2.043 |
|  | CP of r9 at 250 m | -4.23E-05 | 3.50E-06 | -1.65E-05 | 1.005103 | -1.596 |
|  | ED of Cropland at 2500 m | -7.47E-02 | 8.99E-02 | -5.82E-03 | 1.031792 | -0.175 |
| GPA | CA of r6 at 1000 m | 3.79E-03 | 3.41E-02 | 1.94E-02 | 1.186185 | 2.645 |
|  | CP of r7 at 1000 m | -3.58E-05 | 5.82E-05 | 1.18E-05 | 1.01299 | 0.474 |
|  | CP of r9 at 5000 m | -2.45E-06 | 2.43E-06 | -7.65E-08 | 1.193785 | -0.068 |
| SPDR | LC of Cropland at 5000 m | -2.79E-02 | 2.13E-02 | -3.79E-03 | 1.801744 | -0.322 |
|  | CP of r7 at 2500 m | -2.56E-05 | 1.33E-06 | -1.32E-05 | 1.164852 | -1.939 |
|  | CA of r5 at 1000 | -1.71E-03 | 2.74E-03 | 3.11E-04 | 1.706345 | 0.312 |
| LBB | ED of Woodland at 5000 m | -2.03E-02 | 1.18E-01 | 5.41E-02 | 1.634242 | 1.515 |
|  | LC of Cropland at 5000 m | -4.08E-02 | 1.52E-02 | -1.44E-02 | 1.823695 | -0.98 |
|  | CA of r7 at 1000 m | -4.97E-03 | 6.27E-03 | 6.26E-04 | 1.957509 | 0.208 |
|  | CP of r7 at 2500 m | -2.25E-05 | 2.74E-06 | -1.07E-05 | 1.409646 | -1.614 |

Table S3. Landscape properties at different spatial scales influenced the effects of field adjacent habitats.

| **Effect of adjacent habitats** | **Landscape_variables** | **Lower CI** | **Upper CI** | **Standerdized Coefficients** | **VIF** | **t value** |
| --- | --- | --- | --- | --- | --- | --- |
| Pasture on DBM | LC of Woodland at 500 m | -0.18878 | -0.14142 | -0.1651 | 3.208807 | -6.739 |
| Pasture on DBM | CP of r1 at 1000 m | -0.05013 | -0.01001 | -0.02879 | 3.798122 | -1.801 |
| Pasture on DBM | CP of r10 at 1000 m | 0.000763 | 0.000851 | 0.000807 | 1.783146 | 3.924 |
| Pasture on DBM | CP of r9 at 5000 m | -0.0008 | 0.000171 | 4.67E-05 | 1.14176 | 0.723 |
| Pasture on WF | LC of Cropland at 1000 m | -2.03E-02 | 0.010627 | -5.35E-03 | 1.59E+00 | -0.71 |
| Pasture on WF | CA of r9 at 1000 m | -1.07E-03 | 0.001468 | 2.92E-04 | 1.41E+00 | 0.503 |
| Pasture on WF | CA of r8 at 2500 m | -3.87E-06 | 0.000141 | 6.81E-05 | 1.41E+00 | 1.827 |
| Pasture on WF | ED of Woodland at 2500 m | 1.54E-03 | 0.011714 | 6.58E-03 | 2.17E+00 | 2.504 |
| Pasture on GPA | CA of r5 at 1000 m | -0.00101 | 0.000624 | -1.95E-04 | 2.26E+00 | -0.474 |
| Pasture on GPA | ED of Pasture at 1000 m | 0.00121 | 0.00659 | 4.04E-03 | 1.16E+00 | 3.132 |
| Pasture on GPA | ED of Woodland at 1000 m | 0.001268 | 0.004967 | 3.17E-03 | 2.04E+00 | 3.544 |
| Woodland on DBM | CP of r1 at 2500 m | -5.37E-05 | -3.45E-05 | -4.47E-05 | 2.68E+00 | -10.481 |
| Woodland on DBM | CP of r5 at 1000 m | -2.27E-05 | -1.30E-05 | -1.82E-05 | 3.56E+00 | -8.765 |
| Woodland on DBM | LC of Woodland at 5000 m | -5.96E-02 | -4.68E-02 | -5.34E-02 | 3.12E+00 | -16.696 |
| Woodland on DBM | ED of Woodland at 2500 m | 3.07E-03 | 2.16E-02 | 1.34E-02 | 4.40E+00 | 11.982 |
| Woodland on WF | CA of r6 at 5000 m | -4.80E-05 | 5.82E-05 | 3.54E-06 | 1.14E+00 | 0.131 |
| Woodland on WF | CA of r1 2500 m | -3.43E-04 | 0.001038 | 3.34E-04 | 1.07E+00 | 0.944 |
| Woodland on WF | ED of Cropland at 2500 m | -4.29E-03 | 0.001526 | -1.39E-03 | 1.09E+00 | -0.926 |
| Woodland on LBB | LC of Cropland at 5000 m | -8.44E-03 | 0.026809 | 9.63E-03 | 1.032043 | 1.088 |
| Woodland on LBB | ED of Woodland at 500 m | -9.09E-03 | -0.00308 | -6.10E-03 | 1.028259 | -4.032 |
| Brassica crops on WF | ED Cropland at 2500 m | -0.01137 | -0.00655 | -0.00903 | 1.08E+00 | -7.664 |
| Brassica crops on WF | CA of r8 at 1000 m | -0.00013 | 0.000343 | 0.000106 | 1.09E+00 | 0.871 |
| Brassica crops on WF | CA of r1 at 1000 m | -0.00419 | 0.005802 | 0.000775 | 1.075846 | 0.306 |
| Non brassica crops on CAD | ED of Pasture at 5000 m | 3.18E-02 | 7.84E-02 | 5.51E-02 | 1.031581 | 32.282 |
| Non brassica crops on CAD | CP of r1 at 2500 m | -3.42E-05 | -2.70E-05 | -3.06E-05 | 1.050224 | -16.693 |
| Non brassica crops on CAD | CA of r9 at 2500 m | 1.29E-01 | 1.39E-01 | 1.34E-01 | 1.029571 | 52.749 |

Table S4. Statistical summary of landscape composition and edge density

| **Land uses** | **Spatial scales** | | | | |
| --- | --- | --- | --- | --- | --- |
|  | **250 m** | **500 m** | **1000 m** | **2500 m** | **5000 m** |
| **Landscape composition (%)** | | | | | |
| Crop fields | 49.739 ± 13.584 | 52.925 ± 12.584 | 48.341 ± 11.483 | 49.694 ± 9.402 | 55.251 ± 3.890 |
| Pastures | 13.542 ± 8.224 | 13.118 ± 8.142 | 12.511 ± 6.904 | 11.906 ± 6.112 | 11.333 ± 5.530 |
| Woody vegetation | 34.963 ± 15.583 | 33.957 ± 13.879 | 39.117 ± 11.842 | 38.156 ± 9.454 | 36.879 ± 8.300 |
| Water bodies | Nil | Nil | Nil | 0.024 ± 0.015 | 0.536 ± 0.333 |
| **Edge density (m/ha)** | | | | | |
| Crop fields | 209.562 ± 27.338 | 212.798 ± 19.739 | 200.603 ± 21.166 | 220.319 ± 18.916 | 171.064 ± 20.042 |
| Pastures | 50.115 ± 14.863 | 56.101 ± 15.039 | 48.997 ± 11.818 | 48.592 ± 10.060 | 38.047 ± 8.699 |
| Woody vegetation | 156.816 ± 31.935 | 157.081 ± 26.122 | 204.976 ± 22.724 | 185.584 ± 17.408 | 166.188 ± 14.326 |
| Water bodies | Nil | Nil | Nil | 11.551 ± 6.764 | 17.359 ± 8.142 |

Table S5. The abundance of dominated arthropods (on 10 plants) at different positions within the 24 brassica vegetable sites

| **Arthropod taxa** | **Abundance at the field centre (mean ± sd)** | **Abundance at the field margins adjacent to land-uses (mean ± sd)** | | | |
| --- | --- | --- | --- | --- | --- |
|  |  | **Pastures** | **Woody vegetation** | **Brassica crops** | **Non-brassica crops** |
| *Plutella xylostella* | 4.45 **±** 6.73 | 2.16 **±** 5.26 | 0.70 **±** 1.98 | 3 **±** 6.64 | 1.54 **±** 4.52 |
| *Pieris rapae* | 0.58 **±** 1.34 | 0.04 **±** 0.20 | 0.13 **±** 0.61 | 0.16 **±** 0.48 | 0 |
| Whiteflies | 1.95 **±** 2.89 | 0.54 **±** 1.61 | 0.66 **±** 1.71 | 0.41 **±** 1.44 | 1.04 **±** 2.33 |
| *Brevicoryne brassicae* | 1.37 **±** 1.81 | 0.75 **±** 2.26 | 0.66 **±** 1.73 | 0.62 **±** 1.49 | 0.29 **±** 1.08 |
| *Myzus persicae* | 2.37 **±** 3.25 | 0.5 **±** 1.91 | 2.45 **±** 3.18 | 2.20 **±** 3.48 | 1.29 **±** 3.27 |
| Ladybirds | 0.79 **±** 1.31 | 0.87 **±** 1.42 | 0.58 **±** 1.34 | 0.54 **±** 1.25 | 0.25 **±** 0.84 |
| Spiders | 0.58 **±** 1.13 | 0.33 **±** 1.43 | 3.45 **±** 3.51 | 0.45 **±** 0.88 | 0.13 **±** 0.33 |


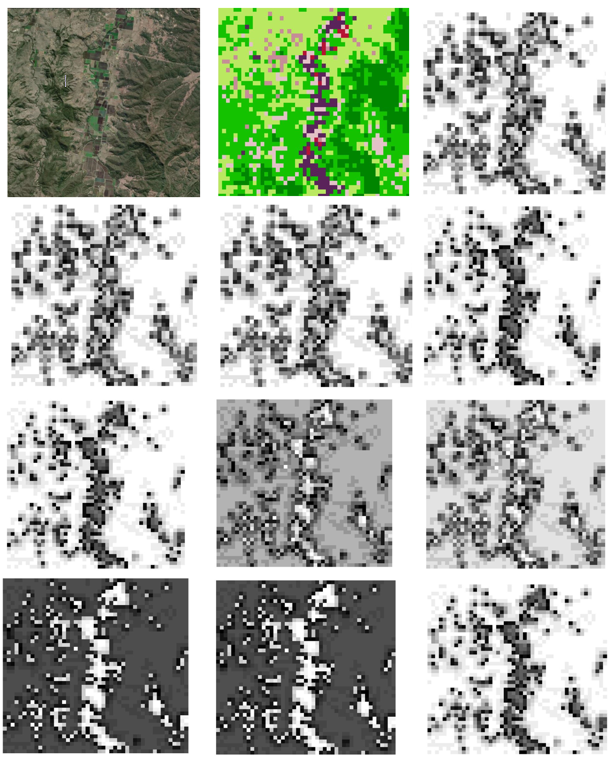


Figure S1. Cost raster cost area metric of cost-distance analysis for site number 14 for all the ten set of cost-ratios with image of the site, image with NDLC dataset. White shades showing favourable land uses and dark shades showing unfavourable land uses.


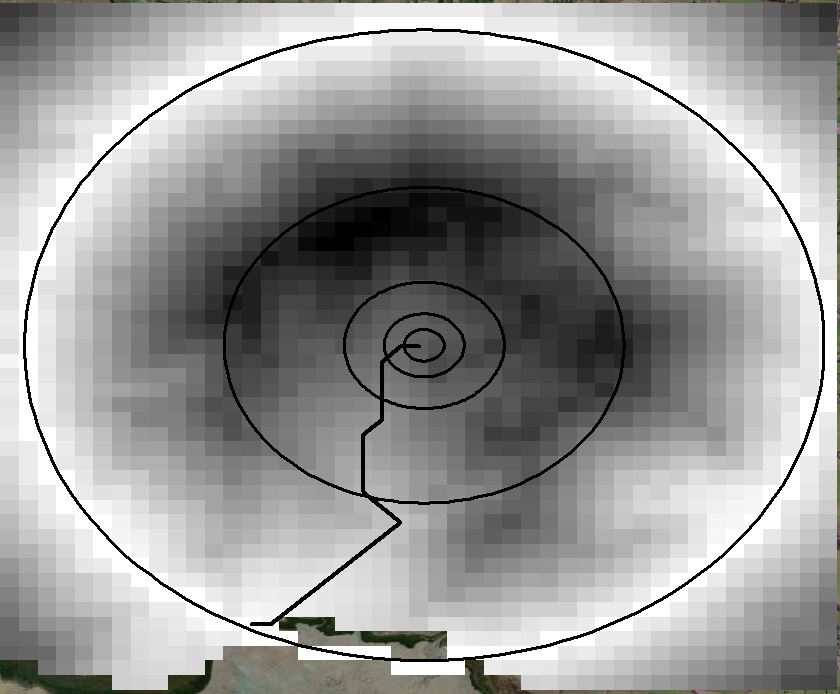


Figure S2. Cost path metric of cost-distance raster of site 9 for cost-ratio r10. Dark shades showing high-cost area, the circles showing five different spatial scales. The line running from centre of the circle to edge of last circle showing the least cost-path to arrive the crop field from a source 5000 m away in the landscape.


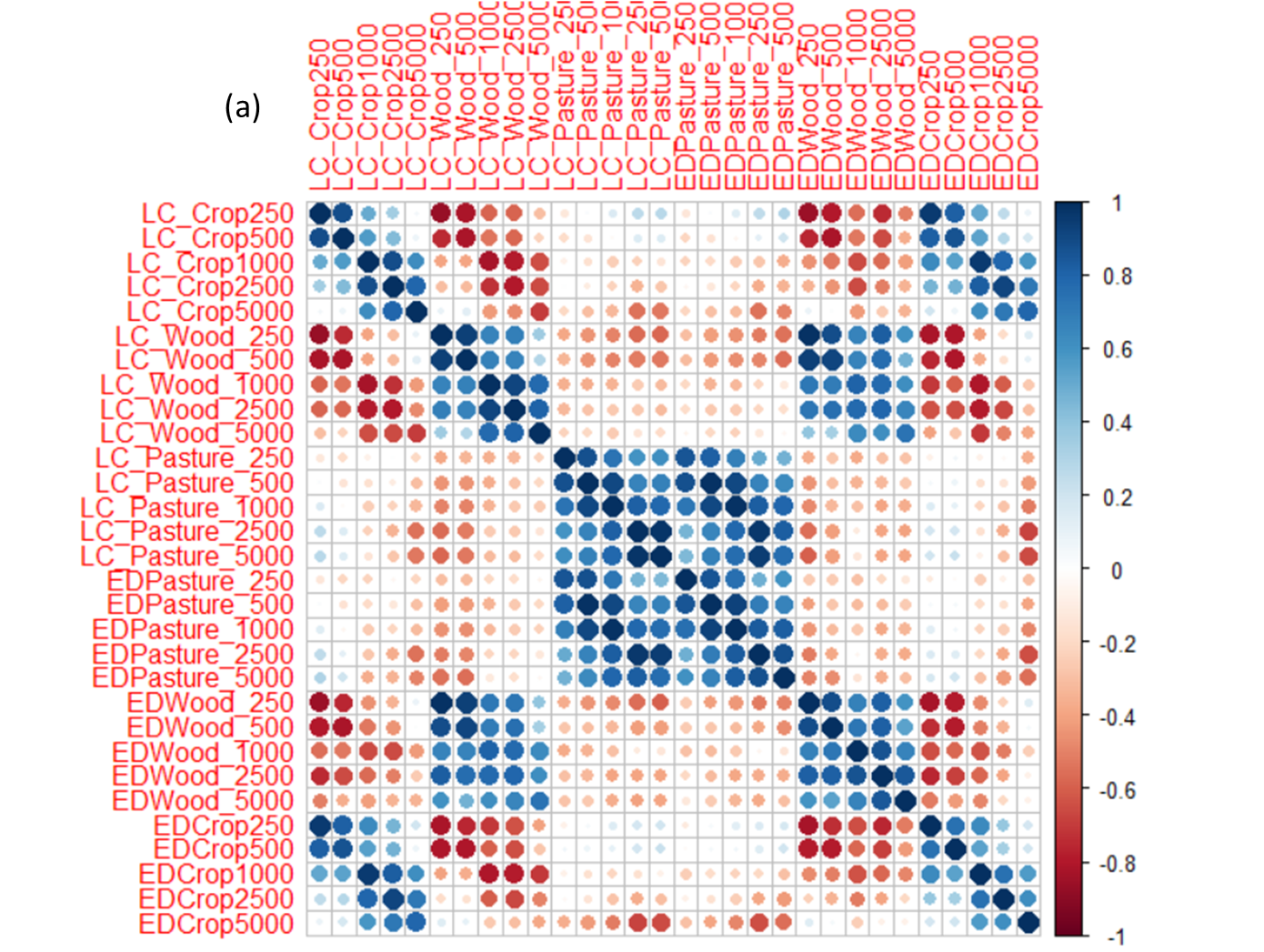


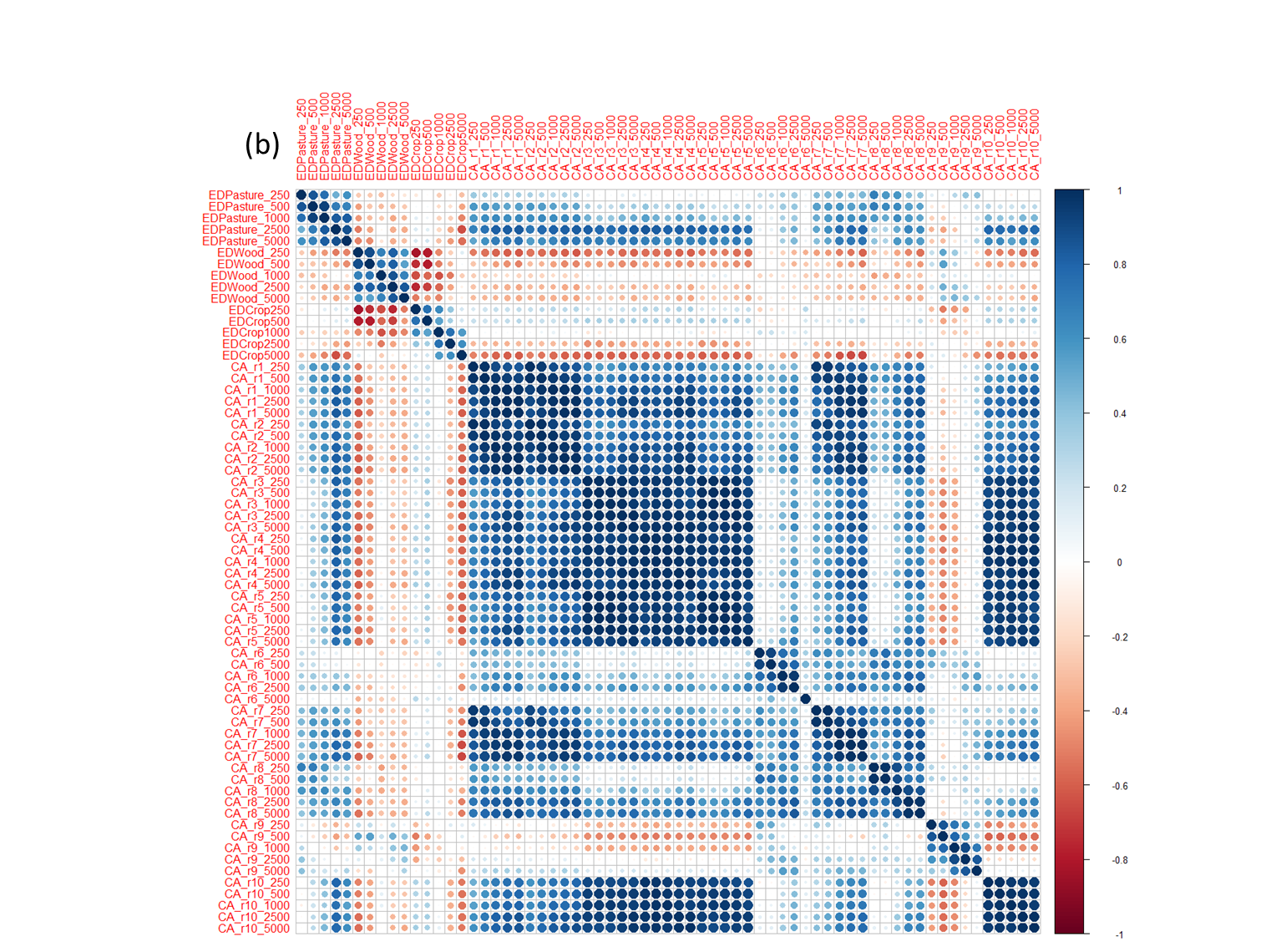


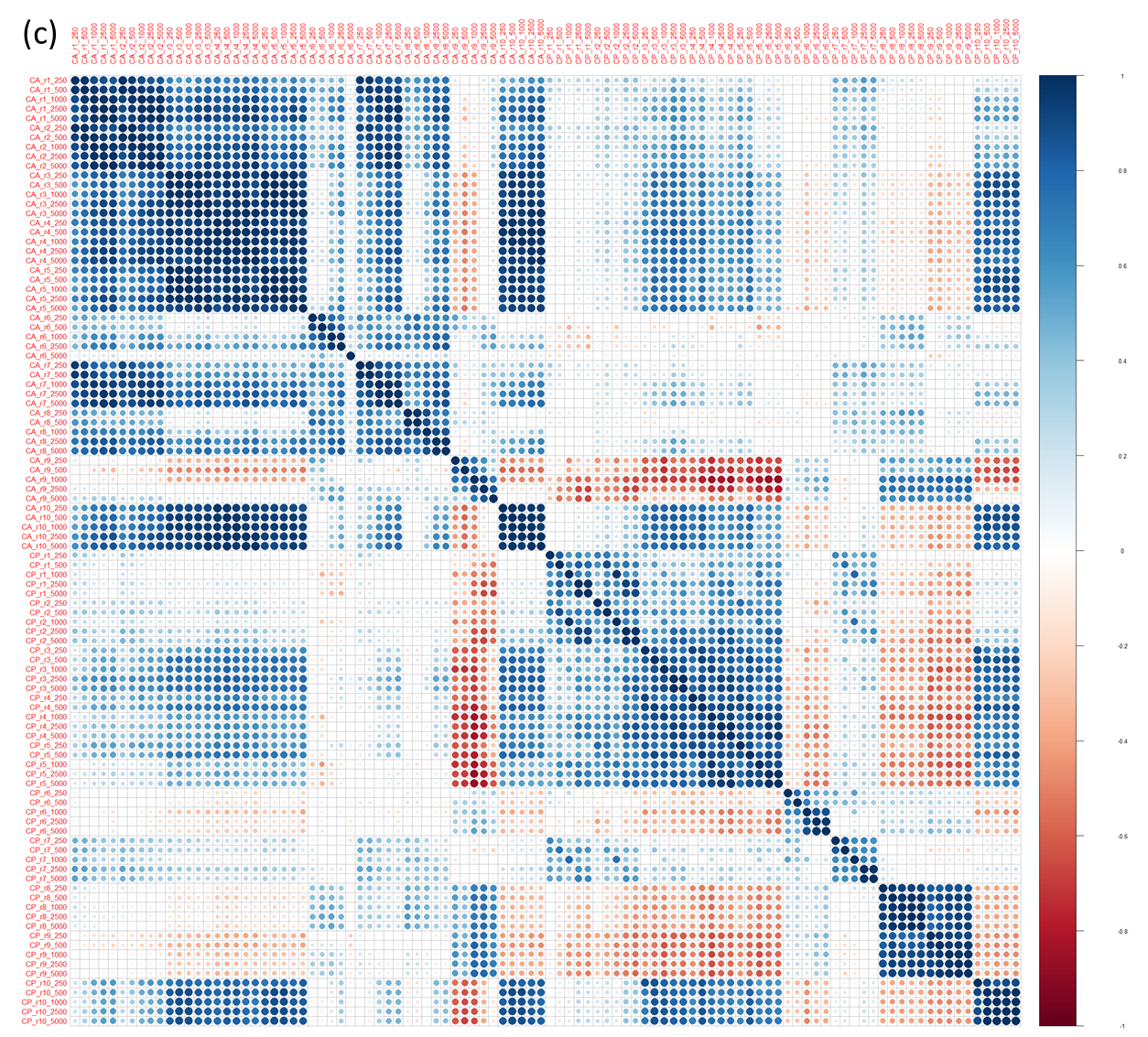


Figure S3. Correlation graphs (a) correlations between the variables of landscape composition and edge density, (b) correlations between the variables of edge density and cost area of landscape connectivity, (c) correlations between the variables of cost area and cost path of landscape connectivity.
